# Neuregulin 3 rs10748842 polymorphism contributes to the effect of body mass index on cognitive impairment in patients with schizophrenia

**DOI:** 10.1038/s41398-020-0746-5

**Published:** 2020-02-11

**Authors:** Yongjie Zhou, Yuhuan Li, Yujie Meng, Jiesi Wang, Fengchun Wu, Yuping Ning, Yi Li, Ryan M. Cassidy, Zezhi Li, Xiang Yang Zhang

**Affiliations:** 1 Research Center for Psychological and Health Sciences, China University of Geosciences, Wuhan, China; 2 Affiliated Wuhan Mental Health Center, Tongji Medical College of Huazhong University of Science & Technology, Wuhan, China; 3 Qingdao Mental Health Center, Qingdao, China; 4 CAS Key Laboratory of Mental Health, Institute of Psychology, Chinese Academy of Sciences, Beijing, China; 5 The Affiliated Brain Hospital of Guangzhou Medical University (Guangzhou Huiai Hospital), Guangzhou, China; 6 Department of Psychiatry and Behavioral Sciences, The University of Texas Health Science Center at Houston, Houston, TX USA; 7 Department of Neurology, Ren Ji Hospital, Shanghai Jiao Tong University School of Medicine, Shanghai, China

**Keywords:** Schizophrenia, Clinical genetics

## Abstract

There is evidence that obesity or higher body mass index is correlated with cognitive impairment in schizophrenia. Recent studies have demonstrated that genetic risk factors, such as the *NRG3*, are correlated with both elevated BMI and reduced cognitive function. In present study, we aimed to determine whether possession of the *NRG3* rs10748842 influences the correlation between elevated BMI and reduced cognitive ability in schizophrenia. To our knowledge, this has never been examined before. A total of 625 inpatients with schizophrenia and 400 controls were recruited. The Repeatable Battery for the Assessment of Neuropsychological Status (RBANS) was performed to assess cognitive function. We used multiple analysis of covariance (MANCOVA), analyses of covariance (ANCOVA), Pearson correlations, partial correlations, and multivariate regression analysis to test the influence of *NRG3* rs10748842 on the aforementioned variables. All RBANS five sub-scores and total score were lower in patients than those in controls (all *p* < 0.001). Patients carrying *NRG3* rs10748842 TC + CC heterozygous genotype had lower attention score compared to TT homozygous genotype (adjusted *F* = 4.77, *p* = 0.029). BMI was positively associated with language score in patients (*β* = 0.387, *t* = 2.59, *p* = 0.01). Interestingly, we further found positive association between BMI and language score in TT carriers (partial correlations: *r* = 0.13, adjusted *p* = 0.004; multivariate regression: *β* = 0.42, *t* = 2.66, *p* = 0.008), but not in CT + CC carrier (*p* > 0.05). Our study demonstrated that *NRG3* rs10748842 was associated with cognitive impairments, especially attention performance in schizophrenia. Moreover, *NRG3* rs10748842 altered the effect of BMI on cognitive impairments as measured by the RBANS language score in chronic patients with schizophrenia.

## Introduction

Cognitive impairment is a core characteristic of schizophrenia, observable ~98% of patients across the domains of memory, attention, language, visuospatial, processing speed, learning, and executive function^1,2^. Cognitive impairments are observed in both first episode and chronic patients with schizophrenia^3^. They are even observable during their prodromal stage and throughout the illness course despite improvements in psychopathological symptoms after pharmacological treatment^4^. Furthermore, cognitive impairment affects treatment outcomes and everyday disability in patients with schizophrenia^5,6^. Thus, it is of interest to see whether there are mechanisms underlying cognitive impairment, which could be intervened upon to improve outcomes—however, these are multifactorial and are as yet unresolved. Genetic variance is one such mechanism and it is well established that certain genes predispose patients to cognitive impairment^7^. The heritability range varies in different domains in patients with schizophrenia, including working memory (0.3–0.6), executive function (0.3–0.6), episodic memory (0.3–0.6), and attention (0.54)^8^. Additional evidence has indicated that cognitive impairment often occurs in unaffected relatives of patients with schizophrenia^9,10^. On the other hand, accumulating research has suggested that non-genetic factors may also affect cognitive impairments in schizophrenia such as lifestyle^11^, vascular risk factors, and metabolic syndrome^12,13^. Obesity and higher body mass index (BMI) are common in patients with schizophrenia^14^. Indeed even outside of schizophrenia, some studies have shown bidirectional effect of BMI on cognitive impairments^15^. Only a few studies have found no association between BMI and cognitive impairments^16,17^. However, the directional relationship has not been established or the effect of genetic risk factors on this relationship. Nor is it known whether schizophrenia is a modifying influence on the relationship between cognition and BMI.

This question is of interest, because multiple studies have demonstrated a role for genetic factors in both cognitive performance and BMI, showing a common genetic effect on BMI and cognitive function^18–20^. For example, Marioni et al.^20^ found that common genetic variants associated with BMI explain a significant proportion of cognitive function variances and vice versa, indicating shared genetic underpinnings for BMI and cognitive function. Frazier-Wood et al.^21^ demonstrated significant shared genetic effects between cognitive performance and BMI in 1,312 twins. Laitala et al.^22^ provided the evidence of the genetic effects on the relationship between midlife BMI and senile cognition in a large sample of 2606 Finnish twins. Thus, this interaction seems like a promising avenue of exploration for both the etiology and therapy of obesity and cognitive impairment. However, to our knowledge, no study has investigated whether genetic factor, BMI and their interactions might affect cognitive function in schizophrenia, and the BMI might influence cognitive performance based on different genetic background.

A prominent hypothesis on the etiology of cognitive impairment and schizophrenia is neurodevelopmental dysfunction, leading to altered basic neurocircuitry^23,24^. Neuregulin 3 (*NRG*3), a neuronal enriched growth factor, plays an important role in neuronal development, including plasticity, development, differentiation, and proliferation^25^. *NRG3* is located on chromosome 10q22-q23; variation in this locus predisposes individuals to schizophrenia as demonstrated by genome-wide studies^26,27^. In addition, *NRG3* mutation has been indicated to affect neurocognitive impairment^28^, especially in patients with schizophrenia^29^.

Strikingly, *NRG3* has also been implicated to be associated with BMI in an Asian population through genome-wide scanning^30^. Therefore, in this study, we sought to determine (1) whether the *NRG3* polymorphism rs10748842 might affect cognitive impairment in schizophrenia; (2) whether BMI might affect cognitive impairments in schizophrenia; and (3) whether *NRG3* polymorphism rs10748842 mediates the effects of BMI on cognitive impairment in schizophrenia in a large sample of Chinese Han population.

## Materials and methods

### Subjects

The present study was reviewed and approved by the Institutional Review Boards of Beijing Hui-Long-Guan hospital. Written informed consents were obtained from all subjects or guardians. The patients recruited in this study satisfied the following inclusion criteria: (1) Han Chinese (2) aged from 18 to 75 years; (3) met the criteria of the Diagnostic and Statistical Manual of Mental Disorders, Fourth Edition (DSM-IV) for schizophrenia; (4) had at least 5 years of illness duration; (5) had been treated with antipsychotics for at least 12 months, primarily with one antipsychotic drug.

A total of 625 patients were recruited with an average age of 45.03 ± 10.19 years. Antipsychotics included clozapine (*n* = 273), risperidone (*n* = 114), quetiapine (*n* = 56), chlorpromazine (*n* = 46), aripiprazole (*n* = 32), sulpiride (*n* = 27), perphenazine (*n* = 19), olanzapine (*n* = 17), haloperidol (*n* = 7), and others (*n* = 34). The antipsychotic doses equivalent to chlorpromazine were 390.35 ± 295.35 mg/day. A total of 400 healthy controls were recruited from the local community. Subjects with any major Axis I disorders or with family history of mental disorders were excluded. All subjects underwent physical examinations and lab tests prior to enrollment. Any subjects with cancer, infections, uncontrolled diabetes, cerebrovascular disease, cardiovascular disease, and pregnancy were excluded. Neither patients nor healthy controls had drug or alcohol abuse/dependence other than smoking. The demographic and clinical profile of subjects were shown in Table 1.

**Table 1:** Demographic profiles in patients with schizophrenia and controls.

Variable	Patients (*N* = 625)	Controls (*N* = 400)	Statistic	*p*
Age (years)	45.03 ± 10.19	44.73 ± 13.50	0.37	0.71
Gender (male)	512 (81.9%)	157 (39.3%)	159.90	<0.0001
Education (years)	8.45 ± 3.65	9.73 ± 5.60	4.06	<0.0001
BMI	23.97 ± 4.45	25.16 ± 4.26	4.20	<0.0001
Onset age	26.60 ± 20.19	−	−	−
Illness duration (years)	21.14 ± 9.90	−	−	−
Atypical antipsychotic (*n*)	439	−	−	−
Typical antipsychotic (*n*)	186	−	−	−
Daily dose (mg/day) (chlorpromazine equivalents)	390.35 ± 295.35	−	−	−
Antipsychotic treatment duration (months)	31.37 ± 46.64	−	−	−
PANSS score		−	−	−
Positive symptoms	11.92 ± 6.26	−	−	−
Negative symptoms	20.14 ± 8.69	−	−	−
General psychopathology	25.15 ± 7.11	−	−	−
Total score	57.20 ± 16.51	−	−	−

### Clinical interview and cognitive assessments

An in-house socio-demographic scale was used to collect relevant information. The Structure Clinical Interview for DSM-IV (SCID-I/P) was used to screen each participant as conducted independently by two psychiatrists. Clinical symptoms of patients were assessed by using the Positive and Negative Syndrome Scale (PANSS)^31^.

Cognitive performance of participants was assessed by using the Repeatable Battery for the Assessment of Neuropsychological Status (RBANS, Form A). We used the translated Chinese version of this test, which has established validity and test–retest reliability in patients with schizophrenia and healthy controls^32^. The RBANS assesses cognitive impairment via a total score and scores for immediate memory, attention, language, visuospatial/constructional, and delayed memory, calculated by age-adjusted index scores through 12 subscales. All patients were in stable state without acute psychotic symptoms^32^.

### DNA extraction and SNP genotyping

DNA was obtained from the peripheral vein blood of each participant through salting-out method and stored in −80 degree^31^. The *NRG3* polymorphism rs10748842 was genotyped by using Matrix-Assisted Laser Desorption/Ionization Time of Flight Mass Spectrometry (MALDI-TOF MS) in the MassARRAY System (Sequenom Inc., San Diego, CA, USA) according to the method described in our previous studies^31^. A randomly selected 5% of samples were repeat-genotyped for quality control; the error rate was 0.1%.

### Statistical analyses

Student’s *t*-test and Chi-square test were used for continuous variables and categorical variables, respectively. The *χ*2 test for goodness of fit was used for Hardy–Weinberg equilibrium in patients and healthy control subjects. Since there was almost no homozygous variant CC genotype in our study (about 0.96% in the patients and 0.25% in healthy controls), we combined the CC and TC genotypes as a group.

To detect the difference in cognitive scores between patients and controls, multiple analysis of covariance (MANCOVA) was applied to investigate the association between genotype and cognition. We set cognition scores as dependent variables, with diagnosis as the fixed factor, and adjusted for sex, BMI, and years of education as covariates. Then the models of covariance (ANCOVA) were applied using the RBANS five subset and total scores as the dependent variables respectively, with the diagnosis as an independent variable, and with sex, years of education, BMI, and smoking as covariates.

To identify the differences in cognitive scores based on different genotypes in the patients or controls, the model of 2 × 2 MANCOVA (genotype × diagnosis) was first used to report the overall p value, and then in this model the main effects of diagnosis, genotype, and genotype × diagnosis were tested. In this MANCOVA model, the diagnosis and the *NRG3* polymorphism rs10748842 genotype were used as the independent variables, with sex, BMI, years of education, and smoking as the covariates. Furthermore, the main effects of diagnosis, genotype, and genotype × diagnosis were also examined through MANCOVA model.

Then, ANCOVA analysis was applied, with age, sex, BMI, education, smoking, as the covariates in control group, together with illness duration, hospitalization times, onset age, antipsychotic types (typical antipsychotics or atypical antipsychotics), daily dose, and antipsychotic duration as covariates in patients. Bonferroni corrections were used in each test for multiple tests. Further, we used multivariate regression analysis (stepwise) with the RBANS five subset and total scores as dependent variables respectively, using *NGR3* genotype as an independent variable in the patients, adjusting for covariates, including age, sex, BMI, education, smoking, illness duration, onset age, antipsychotic types, daily dose, and current antipsychotic medication duration in patients. ANCOVA was also used to identify the main effect of the *NRG3* genotype on BMI.

To investigate the effects of BMI on cognitive performance in patient and control groups, multivariate regression was applied, with the RBANS five subset and total scores as dependent variables, using BMI as an independent variable, adjusting for demographic and clinical covariates in patients and controls, respectively.

Pearson correlations were used to examine whether cognitive function was related to BMI in different genotype groupings in the patient group. Partial correlations were used adjusting for age, sex, years of education, smoking, illness duration, onset age, antipsychotic types, daily dose, and antipsychotic duration. Further, in each *NGR3* genotype group, multivariate regression analysis (stepwise) was used with the RBANS five subsets and total score as dependent variables, using BMI as independent variables, adjusting for covariates including age, sex, education, smoking, illness duration, onset age, antipsychotic types, daily dose antipsychotic duration in patients.

Quanto Software was used to calculate the power of the sample under log additive, recessive and dominant models, setting 1% as the prevalence of schizophrenia in population.

## Results

The demographic and clinical profiles of the patients and healthy controls were detailed in Table 1. Except for age, significant differences in sex, BMI and years of education were found between patients and healthy controls (all *p* < 0.0001), adjusted as covariates in the following analyses. Table 2 shows the demographic and clinical data by *NRG3* rs10748842 genotype in case and control groups, respectively.

**Table 2:** Demographic profiles in different genotypic groups among patients and controls.

Variable	Patients (*N* = 625)				Controls (*N* = 400)			
	TT (*N* = 544)	TC (*N* = 81)	Statistic	*p*	TT (*N* = 347)	TC (*N* = 53)	Statistic	*p*
Age (years)	44.91 ± 10.21	45.84 ± 10.81	0.77	0.44	44.67 ± 13.53	45.15 ± 13.44	0.24	0.81
Gender (male)	447 (82.2%)	66 (81.5%)	0.17	0.92	135 (38.9%)	22 (41.5%)	0.13	0.72
Education (years)	8.42 ± 3.75	8.70 ± 2.93	0.67	0.50	9.94 ± 5.80	8.38 ± 3.86	1.90	0.06
BMI	23.97 ± 4.52	23.99 ± 4.00	0.04	0.97	25.10 ± 4.20	25.50 ± 4.64	0.62	0.53
Onset age	23.76 ± 6.80	30.21 ± 23.17	1.01	0.28	−	−	−	−
Illness duration (years)	21.13 ± 9.95	21.19 ± 9.64	0.05	0.96	−	−	−	−
Daily dose (mg/day) (chlorpromazine equivalents)	390.47 ± 308.32	389.49 ± 187.59	0.03	0.98	−	−	−	−
Antipsychotic treatment duration (months)	31.27 ± 47.27	32.10 ± 42.52	0.15	0.88	−	−	−	−
PANSS score								
Positive symptoms	11.95 ± 6.36	11.67 ± 5.54	0.37	0.71	−	−	−	−
Negative symptoms	19.94 ± 8.62	21.48 ± 9.11	1.48	0.14	−	−	−	−
General psychopathology	25.10 ± 7.11	25.52 ± 7.19	0.49	0.62	−	−	−	−
Total score	56.99 ± 16.56	58.67 ± 16.17	0.85	0.40	−	−	−	−

### Association analysis of NRG3 rs10748842 with schizophrenia

Genotyping was completed in 625 patients and 400 controls. The Hardy–Weinberg equilibrium of *NRG3* rs10748842 genotypes were consistent in both patient and control groups (both *p* > 0.05). The distributions of *NRG3* rs10748842 allele and genotype were not significantly different between patient and control groups (*χ*^2^ = 0.09, *p* = 0.77; *χ*^2^ = 1.91, *p* = 0.39; respectively). Further logistic regression analysis (backward conditional) demonstrated no significant difference in the distribution of *NRG3* rs10748842 allele or genotype between patient and control groups (both *p* > 0.05).

### Cognition between patients and controls

As predicted, MANCOVA showed significant difference in cognitive perform between patient and control groups (Wilks’ lambda *F* = 112.06; *p* < 0.001). Then ANCOVA analysis conducted for each cognitive score showed that all RBANS scores were significantly decreased in patients compare to healthy controls (all *p* < 0.001) (Table 3). All these significant differences survived after Bonferroni correction (all *p* < 0.001).

**Table 3:** Comparisons among the RBANS total and five domain scores by diagnostic and genotypic groups.

RBANS scores	Patients with schizophrenia^a^		Controls		Diagnosis		Genotype		Diagnosis X genotype	
	TT (*N* = 544)	TC + CC (*N* = 81)	TT (*N* = 347)	TC + CC (*N* = 53)	*F*	*p*	*F*	*p*	*F*	*p*
Immediate memory	56.28 ± 15.10	54.24 ± 15.65	76.02 ± 17.07	74.06 ± 20.33	167.34	<0.001	1.71	0.19	0.001	0.98
Attention	61.83 ± 17.70^a^	57.70 ± 13.08^a^	87.87 ± 20.26	84.47 ± 21.43	226.60	<0.001	4.60	0.03^b^	0.04	0.87
Language	73.42 ± 17.54	70.54 ± 16.70	94.15 ± 12.98	92.85 ± 13.96	203.97	<0.001	1.92	0.17	0.28	0.60
Visuospatial/construction	75.02 ± 17.50	71.70 ± 15.94	80.08 ± 15.89	78.06 ± 13.73	13.09	<0.001	2.88	0.09	0.18	0.68
Delayed memory	61.99 ± 18.37	61.96 ± 18.09	86.54 ± 14. 93	85.06 ± 17.48	213.15	0.001	0.21	0.64	0.20	0.66
Total score	59.12 ± 13.38	56.64 ± 11.35	80.41 ± 15.02	78.30 ± 16.70	261.71	<0.001	2.98	0.08	0.02	0.89
BMI	23.97 ± 4.52	23.99 ± 4.00	25.10 ± 4.20	25.50 ± 4.64	10.00	0.002	0.25	0.62	0.20	0.66

^a^A significant genotypic effect on Attention score in patients with schizophrenia.

^b^There was a significant genotype effect on Attention score in all subjects (*F* = 4.60, *p* = 0.03). Patients carrying TC + CC had lower attention score than those carrying CC in patient group (adjusted *F* = 4.77, *p* = 0.029). There was no difference in attention score between controls carrying TC + CC and carrying CC (adjusted *F* = 0.20, *p* = 0.66).

### Effects of NRG3 rs10748842 on cognition in patients and controls

As predicted, MANCOVA analysis showed significant main effect of diagnosis (Wilks’ lambda *F* = 61.54; *p* < 0.001), but no effect of either genotype (Wilks’ lambda *F* = 0.87; *p* = 0.52) or genotype x diagnosis (Wilks’ lambda *F* = 0.32; *p* = 0.93). Then ANCOVA analysis was conducted for each cognitive score shown in Table 3, and a significant genotype effect on attention score was found in all subjects (*F* = 4.60, *p* = 0.03). Further, ANCOVA analysis demonstrated that patients carrying TC + CC had lower attention score than those carrying TT only in patient group (*F* = 4.07, *p* = 0.04). After adjusting for the age, sex, education years, smoking, illness duration, onset age, medication type, dose, and medication duration as the covariates, the difference still remained significant (adjusted *F* = 4.77, *p* = 0.029). However, no difference was found in attention score between TC + CC carrier and TT carrier in control group (adjusted *F* = 0.20, *p* = 0.66). We did not find significant genotype x diagnosis effects on other RBANS scores and total score (all *p* > 0.05) (Table 3).

Multivariate stepwise regression further showed that the genotype of *NRG3* rs10748842 was correlated with the attention score independently only in patient group (*β* = −4.40, *t* = 2.27, *p* = 0.02).

### Effects of NRG3 rs10748842 on BMI in patients and controls

There was a significant main effect of diagnosis on BMI (*F* = 10.00, *p* = 0.002) (Table 3). No genotype or diagnosis × genotype effects on BMI were observed (all *p* > 0.05). As shown in Table 3, there was no difference in BMI between genotypes in the all subjects or when the patients and healthy controls were detected separately (all *p* > 0.05). It suggested that *NRG3* rs10748842 genotype had no effect on BMI in patients with schizophrenia.

### Correlation of BMI with cognitive performance in patients and controls

Multivariate regression showed that BMI was positively associated with language (*β* = 0.387, *t* = 2.59, *p* = 0.01) in patients, but not with other RBANS subscales or total score. There was no association of BMI with any RBANS subscale or total scores (all *p* > 0.05).

### Relationships between BMI and cognitive score: affected by NRG3 rs10748842

**Fig. 1:**
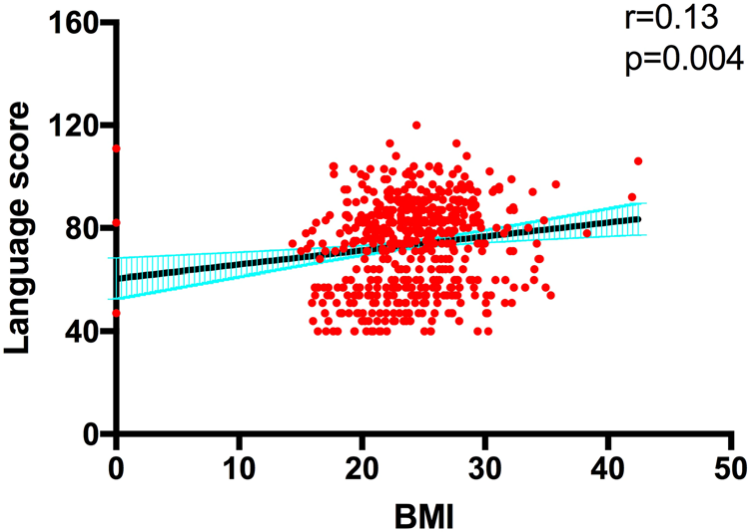
Language score in patients with schizophrenia carrying *NRG3* rs10748842 polymorphism genotype TT. After adjusting for age, sex, years of education, smoking, illness duration, onset age, antipsychotics type, antipsychotics dose (chlorpromazine equivalents), and antipsychotics treatment duration, language score was associated with BMI in patients with TT genotype (*r* = 0.13, *p* = 0.004).

We further explored whether the relationship of BMI with cognitive function differed between genotypic subgroups in patients and healthy controls separately. In patient group, Pearson correlations showed significant positive association between BMI and language score in TT carriers (*r* = 0.14, *p* = 0.001). After adjusting for age, sex, years of education, smoking, illness duration, onset age, antipsychotics type, antipsychotics dose (chlorpromazine equivalents), and antipsychotics treatment duration, partial correlation analysis showed positive association between BMI and language score in patients with TT genotype (*r* = 0.13, *p* = 0.004) (Fig. 1). Further, multivariate regression confirmed that BMI was positively associated with language (*β* = 0.42, *t* = 2.66, *p* = 0.008) in patients carrying TT genotype. However, there was no association between BMI and language performance in patients carrying TC + CC genotype, adjusting for clinical covariates (*r* = 0.02, *p* = 0.83).

In control group, there was no association between BMI and any RBANS subscales, as well as total score in each different *NGR3* genotypic subgroup (all *p* > 0.05), suggesting that there was no significant interaction effect of BMI*NGR3 genotype on cognitive function in the control group.

## Discussion

In the present study, we found that (1) all RBANS scores were decreased in patients with schizophrenia compared to healthy controls; (2) patients with *NRG3* rs10748842 genotype TC + CC had lower attention score than those with TT; (3) BMI was positively associated with the language score in schizophrenia. Interestingly, when stratified by genotype, positive association between BMI and language score was found only in patients with TT genotype.

Cognitive impairment is common in the patients with schizophrenia. Our results were consistent with previous study showing worse cognitive function in patients with schizophrenia than in healthy controls^33^. However, up to date, the underlying mechanisms of cognitive deficits in schizophrenia are unclear. Some studies have indicated that genetic factors are involved in cognitive impairment in schizophrenia^7^. The neurodevelopmental hypothesis has been proposed for the etiology and cognitive impairment in schizophrenia^34,35^. Neuregulin 3 (NRG3) is a member of neuregulin family, which plays an important role in neuronal development, including plasticity, development, differentiation, and proliferation^25^. Previous evidence indicated that *NRG3* gene mutations were correlated with cognitive impairment in patients with schizophrenia^29,36,37^. Further, some of these studies also demonstrated the specific association or opposite association between *NRG3* gene mutations and cognitive inpatients in schizophrenia and healthy controls. For example, Morar et al.^29^ found that *NRG3* mutation might adjust early attentional processes with opposite influence between patients with schizophrenia and healthy controls. Tost et al.^37^ further provided the evidence indicating that *NRG3* polymorphism rs10748842 affected neuronal function in the prefrontal cortex in schizophrenia patients, but this effect was not found in healthy subjects or in healthy siblings of patients. Recent study also showed that *NRG3* rs10748842 was associated with cognitive impairment only in schizophrenia^38^. However, the underlying mechanism of this patient-specific association might be complex and has not been investigated. We speculated that the following aspects might interpret this phenomenon. First, previous evidence has demonstrated that NRG3 can regulate dopaminergic neurotransmission^39,40^. Further, the activation of Neuregulin-ErbB signaling pathway can also mediate the dopamine levels^41^. Second, Bartolini et al.^42^ proved that NRG3 could regulate the final allocation of GABAergic interneurons in the cortex. It has been established that the dysfunction of dopaminergic neurotransmission and GABAergic neurotransmission has been believed to be involved in pathophsiology of schizophrenia^43,44^. On the other hand, dopamine and GABAergic neurotransmission can play a critical role in cognitive functions^45,46^. Thus, taken together, the abnormality of NGR3 might enlarge the dysfunction of dopaminergic neurotransmission and GABAergic neurotransmission in patients with schizophrenia, which can cause more significant cognitive impairment in schizophrenia than in healthy controls.

In the present study, we provided further evidence of genetic effects on cognitive impairment, showing that *NRG3* polymorphism rs10748842 was associated with cognitive impairment, especially attention performance in schizophrenia. *NRG3* polymorphism rs10748842 is located in the non-exonic ultra-conserved genomic elements (UCEs), where critical functional element TAATTA motif exists, and the rs10748842 (T) positively binds with multiple important family of transcript factors, and thus rs10748842 strongly affects and predicts *NRG3* expression in human brain^47,48^. In addition, rs10748842 can affect *NRG3* class II and class III, specifically expressed in brain^49^. Kao et al.^48^ demonstrated that *NRG3* expression in patients with schizophrenia carrying rs10748842 C allele was 60% lower than those in patients carrying the T allele. Paterson et al.^49^ also observed in both bipolar disorder and depression that NRG3 levels of isoforms II and III were lower in patients with heterozygous TC genotype than those in T allele carriers.

The impact of BMI on cognitive function is a public health problem in adolescence and adulthood, but the results are still inconsistent. Recently Olivo et al.^50^ demonstrated worse cognitive function in overweight and obese individuals from the UK Biobank cohort with a sample of 170,310 participants. However, Qizilbash et al.^51^ reported that lower BMI in middle-aged and elderly people was found to have higher risk of dementia in cohort of 1,958,191 people. Similarly, till now, the high prevalence of metabolic syndrome—especially obesity and higher BMI—in patients with schizophrenia has also received increasing attention. The relationship between BMI and cognitive impairment in schizophrenia has been recognized, but the results were also inconclusive^12^. Some studies have shown poor cognitive performance in schizophrenia patients with higher BMI. For example, Kimhy et al.^52^ showed that BMI was inversely associated with cognitive performance. Rashid et al.^13^ indicated that BMI could be used as a potential predictor of higher risk of cognitive impairment in schizophrenia patients. Hidese et al.^53^ recently reported that BMI was negatively associated with cognitive performance in schizophrenia patients. Friedman et al.^16^ demonstrated that a BMI above 25 kg/m^2^ produced negative effects on cognition in both patient group and control group^16^. However, some studies showed no association between BMI and cognition. For example, Takayanagi et al.^17^ found no assocaitation between BMI and cognitive impairment in samples of 1289 individuals with schizophrnia^16^. In addition, some studies have shown a bidirectional effect of BMI on cognitive impairment^15^. Taken together, the above-mentioned findings indicate that the effects of BMI on cognitive impairment in schizophrenia and general population are far more complicated than expected. Other factors might contribute to the impacts of BMI on cognitive impairments in patients with schizophrenia.

Lines of evidence shows shared genetic effects on BMI and cognitive function, and genetic correlation quantified the genetic variants for BMI associated with genetic variants for cognitive function^20^. Relative genes associated with both cognitive function and BMI may have overlapping expression in the anatomical parts of the brain^19^. For example, Marioni et al.^20^ reported that genetic variants associated with better cognitive performance were associated with lower BMI. Frazier-Wood et al.^21^ demonstrated around 30% of the genetic correlation between cognitive performance and BMI^21^. Laitala et al.^22^ showed 12% additive genetic correlation between cognitive performance in the elderly and midlife BMI. Taken together, the above findings suggest shared genetic pathway for BMI and cognitive function. Interestingly, *NRG3* gene was reported to be associated with BMI through genome-wide association study in Asian population^30^. Thus, we reasonably investigated whether BMI affected cognitive impairment in schizophrenia was associated with *NRG3* polymorphism rs10748842. We found that BMI was positively associated with language score in patients with schizophrenia. More interestingly, when performing genotypic stratification analysis, we found a positive association between BMI and language score only in TT patients, but not in CT + CC genotypes, indicating that the interaction between *NRG3* and BMI might affect cognitive function in schizophrenia. It is likely that the BMI might influence cognitive performance based on different *NRG3* genotypes. Previous findings on the impact of BMI on cognitive impairment were inconsistent, probably because they did not consider other interactions, such as genetic factors. To our best knowledge, the current study is the first to provide evidence for the interaction of gene and BMI in cognitive impairment of schizophrenia.

However, the underpinning of the correlation between *NRG3* gene and BMI have been suggested. Guénard et al.^54^ found that the *NRG3* gene associated with obesity traits was correlated with dietary pattern, suggesting that it was involved in metabolic responses to dietary patterns of obesity and glucose metabolism. Meanwhile, the association between BMI and cognition has also been implicated. Some studies indicated that the neurochemical mediators induced by adipocyte could regulate neuronal function directly^55^. On the other hand, leptin predominantly induced by adipocytes could affect appetite and contribute to cognition^56^. Additionally, higher leptin levels have been reported to be correlated with higher BMI^57^, and leptin could enhance synaptic long-term potentiation and plasticity^58,59^. However, further mechanism on interplay of *NRG3* and BMI in cognitive impairments should be investigated in future.

There were some limitations that should be concerned to carefully interpret our results. First, the patients recruited in the present study had chronic schizophrenia, thus it could not represent this influence in first episode patients. However, some studies have indicated that cognitive impairment did not change within the same patient over time, and they could remain relatively stable. Further, cognitive impairment was independent of changes in clinical status, and the pattern and severity of cognitive impairment were generally stable in different clinical conditions of patients^60^. Second, the patients recruited in present study were treated with different antipsychotics, which may affect cognitive function, although they were equivalently converted to chlorpromazine and controlled in the statistical analyses. Third, the results of our study might not be interpreted in other ethical population. Last but not the least, the association of *NG3* rs10748842 with cognition was only marginal, and the effect was only seen in patient group. Thus, there is a need to replicate these findings in additional independent sample, including different situation or stages of the patients. For example, first episode patients, drug naive patients or patients treated with one certain antipsychotic medicine, or other ethical populations.

In conclusion, our study demonstrated that genetic factor *NRG3* rs10748842 might be associated with cognitive impairment, especially attention performance in schizophrenia. Further, BMI might affect cognitive function under certain *NRG3* rs10748842 genotype in schizophrenia, suggesting that *NRG3* rs10748842 might contribute to the effects of BMI on cognitive impairment in chronic patient with schizophrenia.

## Data Availability

All the data are available by requirement from the corresponding author.
